# Microbial Bebop: Creating Music from Complex Dynamics in Microbial Ecology

**DOI:** 10.1371/journal.pone.0058119

**Published:** 2013-03-06

**Authors:** Peter Larsen, Jack Gilbert

**Affiliations:** 1 Biosciences Division, Argonne National Laboratory, Argonne, Illinois, United States of America; 2 Department of Ecology and Evolution, University of Chicago, Chicago, Illinois, United States of America; King Abdullah University of Science and Technology, Saudi Arabia

## Abstract

In order for society to make effective policy decisions on complex and far-reaching subjects, such as appropriate responses to global climate change, scientists must effectively communicate complex results to the non-scientifically specialized public. However, there are few ways however to transform highly complicated scientific data into formats that are engaging to the general community. Taking inspiration from patterns observed in nature and from some of the principles of jazz bebop improvisation, we have generated Microbial Bebop, a method by which microbial environmental data are transformed into music. Microbial Bebop uses meter, pitch, duration, and harmony to highlight the relationships between multiple data types in complex biological datasets. We use a comprehensive microbial ecology, time course dataset collected at the L4 marine monitoring station in the Western English Channel as an example of microbial ecological data that can be transformed into music. Four compositions were generated (www.bio.anl.gov/MicrobialBebop.htm.) from L4 Station data using Microbial Bebop. Each composition, though deriving from the same dataset, is created to highlight different relationships between environmental conditions and microbial community structure. The approach presented here can be applied to a wide variety of complex biological datasets.

## Introduction

In a recent survey, more than two thirds of Americans surveyed reported believing that there is no strong agreement among environmental scientists on the existence and causes of global warming [1]. The scientific community has an opportunity, if not an obligation, to find new and engaging ways to convey ecological science to the public. We propose an approach by which music can be used as public outreach to engage the public in microbial ecology. Repeating patterns and complex interactions between microbial taxa and their environment have been observed in the seasonal variation of microbial communities (e.g. [2], [3], [4]). The principle of repetition is also observed in musical composition [5]. Here, we combine music and biology to generate musical compositions from microbial ecology data.

Previous efforts to convert biological data into music, such as converting the amino-acid sequence of proteins into music (e.g. [6]), or converting genomic DNA sequences into music (e.g. [7]), have generally converted a linear sequence of biological data directly into a musical score. However, while potentially informative, the result of such a simple linear transformation is unable to convey relationships between multiple forms of data. To generate aesthetic musical compositions that interpret the relationships between elements in large biological datasets, we drew inspiration from bebop jazz. The amount of biological information that can be encoded in a single measure of music is considerable. For example, given two chords per measure with a possible selection of eight chords, a melody of six notes from a one-and-a-half octave range per measure, 12 possible patterns of note durations, and three possible patterns of percussion, there are 6.88×10^109^ possible unique measures, a number that far exceeds the estimated number of stars in the universe. Any complex biological experiment can be interpreted as many unique musical compositions, producing an almost unlimited number of possible transformations of data into music.

## Materials and Methods

**Figure 1:**
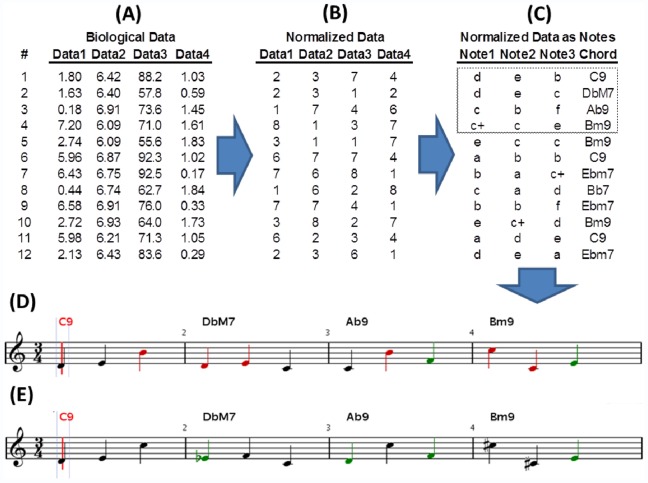
Microbial Bebop method summary. Using a small, hypothetical dataset (A), the general approach to Microbial Music is summarized. Data is first normalized to integer values (B). In this example, values between 1 and 8 were selected. Each integer value is mapped to a specific note or chord (C). The first four observations in (C), identified by dashed outline, are converted into four bars of musical notation in 3/4 time in (D). Chord notes are black, color tones are green, and tones that are dissonant to chord are red. Melody is rectified to harmony in (E). For implementation of rectifying melody to harmony, the freely available jazz improvisation program ‘ImproVisor’ (www.cs.hmc.edu/~keller/jazz/improvisor/) [11] was used. Additional information can be included in resulting composition by incorporating note duration, instrument selection, or tempo as described in ‘Sample Compositions’.

**Figure 2:**
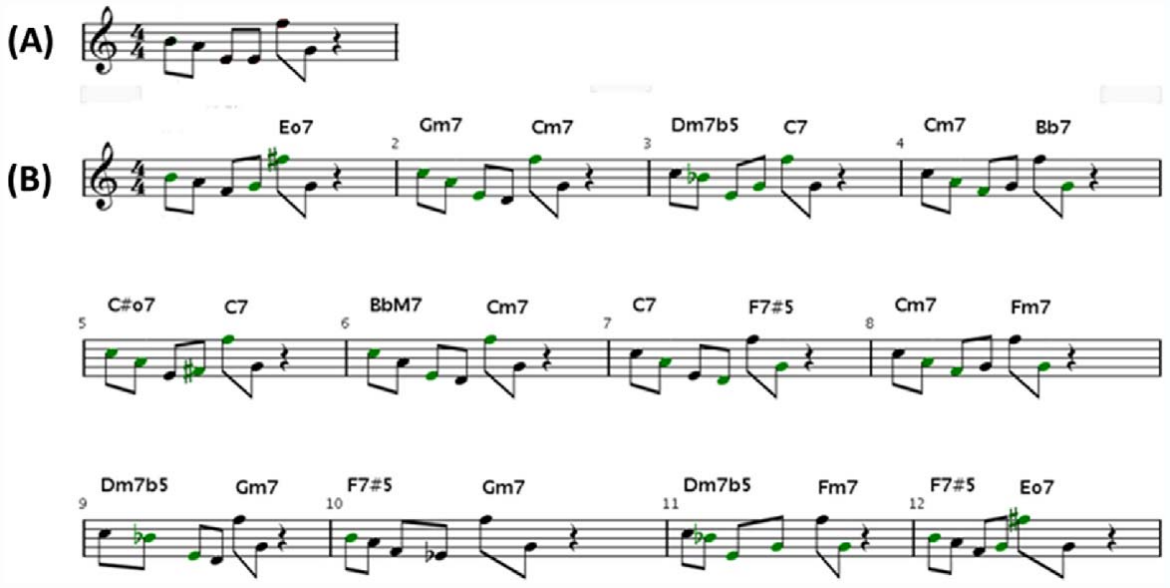
Microbial Bebop highlights relationships between data. In this example, the notes in measure in (A) are comprised of six hypothetical data points. All twelve measures in (B) are derived from the same six data points, but each measure is rectified to different chords, representing the same data played in context of different hypothetical parameters. Each measure in (B) is subtly but audibly distinct, demonstrating ability of Microbial Bebop to represent data in a way that can potentially be interpreted by a listener. An audible version of (B) is available (www.bio.anl.gov/MicrobialBebop.htm).

Bebop, very loosely defined, is characterized by an improvised solo over a specific chord progression [8]. For Microbial Bebop, the melody represents one set of elements from a biological dataset, the chord progression another set of elements. If a single musical measure represents one experimental observation, then a complete composition is generated from all observations in an experiment. The complete procedure for converting numerical data into sound is outlined in **Figure 1**. While the method as outlined only considers pitch and harmony, additional biological data can be translated and incorporated by changing patterns of note duration, changes in chord patterns, or changes in percussion. Specific examples of these are described below. The main advantage of Microbial Bebop over previous efforts to transform scientific data in music is the ability to highlight relationships between data types. The same melody, generated from the same set of data, sounds different when played in the context of chords generated from different data types (**Figure 2**). For example, the melody generated from microbial abundance data played to chords generated from phosphorus concentration data will be audibly distinct from the same abundance data played to chords generated from temperature data.

Although there are numerous possible data sets that can be converted into musical compositions, as an example, a single large dataset from a long-term study of marine microbial populations in the Western English Channel was used [2]. This data is comprised of 72 consecutive monthly 16S rRNA V6 pyrosequencing amplicon datasets and accompanying environmental parameter data collected at the L4 Station between January 2003 and December 2008 (www.westernchannelobservatory.org.uk/). To highlight the ability of Microbial Bebop to generate multiple compositions from a single dataset, such as one from the L4 Station, four algorithmically-generated compositions were produced. Each composition focuses on different aspects of the ecological interactions for the microbial communities of the Western English Channel. The combinations of data used to generate specific examples of Microbial Bebop are described below, and the MP3-formatted versions of compositions can be found at www.bio.anl.gov/MicrobialBebop.htm.

## Results

### Blues for Elle

This composition highlights seasonal patterns in marine physical parameters at the L4 Station. The chords are generated from seasonal changes in photosynthetically active radiation. The melody of each measure is comprised of eight notes, each mapped to a physical environmental parameter, in the following order: temperature, soluble reactive phosphate, nitrate, nitrite, saline, silicate, and chlorophyll A concentrations.

### Bloom

Some marine microbial taxa are most often present in the L4 Station community at very low abundance, but occasionally become highly dominant community members. To link these microbial blooms to relevant physical parameters, the chords in this composition are derived from changes in chlorophyll A concentrations and salinity. The melody for each measure is derived from the relative abundances of typically rare taxa that were observed to occasionally bloom to higher abundance in the following order: Cyanobacteria, *Vibrionales, Opitulates, Pseudomondales, Rhizobiales, Bacillales, Oceanospirallales,* and *Sphingomonadales.*

### Far and Wide

Microbial species of the Order *Rickettsiales*, such as the highly abundant, free-living planktonic species *Pelagibacter ubique*, are typically, highly abundant taxa in L4 Station data. Its relative abundance in the microbial community at L4 Station follows a distinctive seasonal pattern. In this composition, there are two chords per measure, generated from photosynthetically active radiation measurements and temperature. The melody of each measure is six notes that describe the relative abundance of the Order *Rickettsiales*. The first note of each measure is from the relative abundance at a time point. The next five notes of a measure follow one of the following patterns: a continuous rise in pitch, a continuous drop in pitch, a rise then drop in pitch, or a drop then rise in pitch. These patterns are matched to the relative abundance of *Rickettsiales* at the given time point, relative to the previous and subsequent time points. The pattern of notes in a measure is mapped to the relative abundance of *Rickettsiales* with fewer rests per measure indicating higher abundance. For time points at which *Rickettsiales* was the most abundant microbial taxa, the corresponding measure is highlighted with a cymbal crash.

### Fifty Degrees North, Four Degrees West

All of the data in this composition derives from twelve observed time points collected at monthly intervals at the L4 Station during 2007. The composition is composed of seven choruses. Each chorus has the same chord progression of 12 measures each in which chords are derived from monthly measures of temperature and chlorophyll A concentrations. The first and last chorus melodies are environmental parameter data as in ‘Blues for Elle’. The melody in each of the second through sixth chorus is generated from the relative abundances of one of the five most common microbial taxa: *Rickettsiales*, *Rhodobacteriales, Flavobacteriales,* Cyanobactera, and *Pseudomondales*. A different ‘instrument’ is used to represent each microbial taxon. Melodies for microbial taxa were generated as in ‘Far and Wide’.

### Conclusions

If sound policy decisions are to be made regarding response to climate change, scientists will have to engage the non-specialized public in dissemination and discussion of complex data. We propose Microbial Bebop as one approach to involve the non-scientific community in ecological science. While the principle intent of Microbial Bebop is to provide a novel way to symbolize the complex interactions within a microbial community and between microbial taxa and their environment, it is also conceivable that it may have additional applications. The power of harnessing human intuitive understanding to solve biological problems intractable to computational analysis has already been demonstrated (e.g. [9]). Perhaps transformations of complex biological data into music will become an analysis approach that takes advantage of natural human pattern recognition abilities to detect subtle differences in music [10] to similarly leverage crowd sourcing for the analysis of highly complex biological systems. While microbial ecology data was used, there are many possible sources of biological data that can be similarly transformed into music. The possible permutations of data transformed into music are nearly infinite.
